# Somatic Mutations Favorable to Patient Survival Are Predominant in Ovarian Carcinomas

**DOI:** 10.1371/journal.pone.0112561

**Published:** 2014-11-12

**Authors:** Wensheng Zhang, Andrea Edwards, Erik Flemington, Kun Zhang

**Affiliations:** 1 Department of Computer Science, Xavier University of Louisiana, New Orleans, Louisiana, United States of America; 2 Tulane Cancer Center, Tulane School of Medicine, New Orleans, Louisiana, United States of America; Baylor College of Medicine, United States of America

## Abstract

Somatic mutation accumulation is a major cause of abnormal cell growth. However, some mutations in cancer cells may be deleterious to the survival and proliferation of the cancer cells, thus offering a protective effect to the patients. We investigated this hypothesis via a unique analysis of the clinical and somatic mutation datasets of ovarian carcinomas published by the Cancer Genome Atlas. We defined and screened 562 macro mutation signatures (MMSs) for their associations with the overall survival of 320 ovarian cancer patients. Each MMS measures the number of mutations present on the member genes (except for TP53) covered by a specific Gene Ontology (GO) term in each tumor. We found that somatic mutations favorable to the patient survival are predominant in ovarian carcinomas compared to those indicating poor clinical outcomes. Specially, we identified 19 (3) predictive MMSs that are, usually by a nonlinear dose-dependent effect, associated with good (poor) patient survival. The false discovery rate for the 19 “positive” predictors is at the level of 0.15. The GO terms corresponding to these MMSs include “lysosomal membrane” and “response to hypoxia”, each of which is relevant to the progression and therapy of cancer. Using these MMSs as features, we established a classification tree model which can effectively partition the training samples into three prognosis groups regarding the survival time. We validated this model on an independent dataset of the same disease (Log-rank p-value <2.3×10^-4^) and a dataset of breast cancer (Log-rank p-value <9.3×10^−3^). We compared the GO terms corresponding to these MMSs and those enriched with expression-based predictive genes. The analysis showed that the GO term pairs with large similarity are mainly pertinent to the proteins located on the cell organelles responsible for material transport and waste disposal, suggesting the crucial role of these proteins in cancer mortality.

## Introduction

Ovarian cancer is the fifth-leading cause of cancer death among women in the United States [1]. The disease is often called a “silent killer” since its occurrence is usually not detected until an advanced stage. About 70% of the deaths occur in patients with advanced-stage, high-grade serous ovarian carcinomas [2]. The mortality has not been significantly improved in the past three decades [3]. Except for the detection delay and inaccessible location of the ovaries, other factors accounting for the persistent mortality include the poor understanding of the underlying biology and a lack of reliable biomarkers [4].

The formation of tumors largely results from cell growth that gets out of control [5]. In the human genome, there are many different types of genes that control cell growth in a very systematic, precise way. When these genes have an error in their DNA codes, the RNA or proteins that they encode may not function properly. Typically, a series of several mutations to certain classes of genes is usually required before a normal cell will transform into a cancer cell [6]. Nevertheless, some observed mutations may be neutral or even beneficial to patient survival. This perception can be considered from at least two perspectives. First, some mutations may be deleterious to the growth and proliferation of cancer cells, thus offering a protective mechanism to the patients. Second, some mutations may include the actual causal factors for relatively less-malignant subtypes of the same disease. For example, previous studies showed that cases with BRCA1/2 mutations have better overall survival than those with wild type BRCA1/2 in patients with ovarian carcinoma [7], [8].

To date, the Cancer Genome Atlas (TCGA) [9] has generated and released comprehensive genomic, epigenomic and proteomic data of clinically annotated high-grade serous ovarian carcinomas (Ov-HGSCs). These rich data provide an unprecedented opportunity to investigate the genetic mechanisms underlying the variance in the survival of cancer patients and to advance the clinical prognosis and therapy of the disease. Besides the BRCA1/2 genotypes, the TCGA ovarian cancer paper [7] showed that gene expression-based sample clusters are also associated with the survival outcomes. Moreover, recent years have witnessed numerous studies that focus on the re-analysis of the TCGA data. In these works, miscellaneous predictive signatures for survival outcomes have been identified. These signatures include the expression measures of coding and miRNA genes [10], genotypes of germline single-nucleotide polymorphisms (SNPs) [11], methylation patterns of genes in key cancer pathways [12], DNA copy number variations (CNV) [13] and the occurrences of chromosome aberrations [14].

As shown in [7], most of the Ov-HGSCs had 8 to 209 somatic mutations. These mutations, detected by exome sequencing, were present in 8945 genes, and 92% of them were validated by experiments using alternative technologies. However, most of the observed variants may be passenger mutations not involved in the formation and progression of ovarian cancer. Hidden among observed mutations are the individual-specific tumor drivers and the genetic alterations positively or adversely impacting the growth and survival of cancer cells. The identification of the clinically important mutations (genes) is far from completed. A major challenge impeding the effective statistical analysis of the somatic mutation spectrum (SMS) is the data sparseness issue. This is particularly implied by the fact that, among the 510 consensus cancer genes collected in the Catalogue Of Somatic Mutations In Cancer database [15], only six are significant in terms of their mutation frequencies over the 326 tumors. Nevertheless, two recent studies have demonstrated the potential to train a predictive model for survival outcomes of ovarian cancer patients using SMS [16], [17]. In this study, we conducted a unique analysis of the recently updated TCGA's clinical and SMS datasets of ovarian cancer. Our study provides significant insights into the treatment of ovarian cancer and may open novel avenues for molecular prognosis and prediction.

## Results

### Predictive macro mutation signatures for patient survival

We developed a novel method to unravel the relationships between the somatic mutations and the survival time of cancer patients. First, by assuming that the DNA alterations on the genes of a similar function may have equivalent or complementary impacts on the growth and proliferation of cancer cells, we defined 562 macro mutation signatures (MMS), each of which corresponds to a highly-specific Gene Ontology (GO) term with 50 to 500 member genes. For each patient (i.e. a carcinoma sample), the MMS quantities were calculated as the number of the mutations on the genes (except for TP53) covered by the cognate GO term. When a gene involves in multiple GO terms, the mutation(s) present on each gene were counted with respect to each cognate MMS. In this way, we circumvented the sparsity issue inherent to the raw somatic mutation data (see Introduction section). After that, the MMSs were screened for their associations with the overall survival (OS) months of the cancer patients. More specifically, the associations were evaluated by performing the Log-rank test and Cox Proportional Hazards (Cox-PH) regression analysis on the mutation and clinical datasets of 320 training samples. In the implementation, quantities of the MMSs were capped by a ceiling value of 2, which represented that a tumor had at least two mutations present on the member genes covered by the corresponding GO term. Capping the MMS values was performed to alleviate the influence of leverage data points, which were related to un-ordinarily high MMS values and usually occurred in highly-specific GO terms. In the Cox-PH analysis, along with a focused MMS, the ages of the patients at the initial diagnosis and a binary measurement variable indicating the presence of somatic mutation on TP53 gene, which had a modestly significant (p<0.05) effect on the patient survival as shown in our preliminary analysis of the same data, were included as covariates. In the Log-rank test, the three possible values (0, 1, 2) of a specific MMS were factorized as the indicators of three groups.

**Figure 1:**
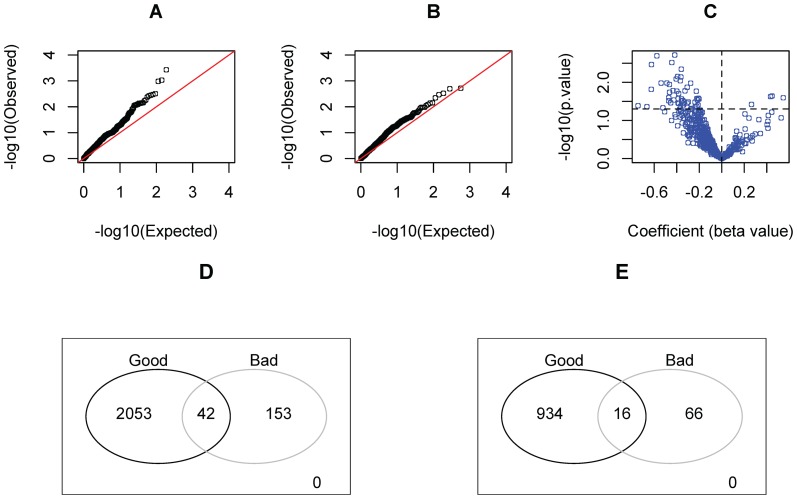
The profile for the associations between the somatic mutations and survival time of patients with ovarian cancer. A (B): The Q-Q plot of the p-values from Log-rank test (Cox-PH regression) for the 562 considered MMSs. C: The volcano plot of the Cox-PH p-values and regression coefficients for the 562 considered MMSs. The horizontal dot line marks p = 0.05. D: The Venn diagram for the entire set of genes covered by the 22 selected MMSs. Specifically, the good (bad) genes are the genes involved in the GO terms corresponding to the 19 (3) positive (negative) MMSs which predict good (poor) clinical outcomes. A gene can belong to both the positive and negative MMSs, therefore may be double counted. E: The Venn diagram for the subset of the genes which are covered by the 22 selected MMSs. Each of the genes has the mutation burden in at least one training sample.

The analysis of the training set (N = 320) demonstrated strong evidence for the existence of an association between the MMSs and survival outcomes. As shown in Figure 1-A and 1-B, the distributional profiles of the p-values (from both the Log-rank test and Cox-PH regression) for the MMSs are deviated from a uniform distribution U (0, 1). Interestingly, most of the regression coefficients (i.e., beta values), especially those corresponding to small p-values, are negative (Figure 1-C). In the Cox-PH model, a negative regression coefficient indicates that the hazard function decreases (or equivalently, survival time increases) as the quantity of the corresponding predictive variable increases [18]. In this regard, we concluded that somatic mutations favorable to the survival of cancer patients are predominant in ovarian carcinoma compared to those indicating poor clinical outcomes. As shown in Figure 1-D and 1-E, this statement is also valid in terms of the number of the involved GO terms and the sizes of the relevant gene sets (Table S1).

Neither Log-rank test nor Cox-PH regression analysis are perfect for evaluating the associations between a MMS and the clinical outcome. The former ignores the patients' ages at the initial diagnosis, which intuitively influence survival time. The latter assumes that the quantity of the hazard functions is linearly dependent on the preprocessed MMS values, which is not true in many cases. Therefore, we determined the top significant MMSs (GO terms) by an alternative method. That is, we selected 20 MMSs if (1) their p-values from both the Log-rank test and Cox-PH analysis are less than 0.05 and (2) the resulting composite p-value (see Method section) is less than 0.025. Among those MMSs, 19 are “positive” predictors for survival time. Considering that the selection criteria may be too stringent for the potential MMSs adversely affecting overall survival outcomes, we chose another two MMSs. These two “negative” predictors, with Log-rank p-values less than 0.01, are relevant to two small patient sets of poor survival and correspond to GO:0045666 and GO:0042393, respectively. In this way, we established a predictor set consisting of 22 MMSs (Table 1).

**Table 1:** The summary of significant MMSs for the overall survival of patients with Ov-HGSCs.

GO (MMS) ID	β	Cox-PH p-value	Log-rank p-value	CP	N1	N2	GO Name
Positive predictors							
GO:0000786	−0.67	4.4E-02	4.3E-05	1.4E-03	65	25	nucleosome
GO:0005765	−0.28	1.1E-02	3.7E-04	2.0E-03	232	135	lysosomal membrane
GO:0050900	−0.42	1.9E-03	3.8E-03	2.7E-03	110	92	leukocyte migration
GO:0007229	−0.44	3.0E-03	8.0E-03	4.9E-03	77	80	integrin-mediated signaling pathway
GO:0010923	−0.54	1.1E-02	3.1E-03	5.7E-03	51	49	negative regulation of phosphatase activity
GO:0007584	−0.63	3.4E-03	1.1E-02	6.1E-03	67	54	response to nutrient
GO:0007067	−0.36	4.5E-03	8.5E-03	6.2E-03	245	126	mitosis
GO:0007160	−0.41	8.2E-03	7.0E-03	7.6E-03	79	75	cell-matrix adhesion
GO:0000910	−0.58	2.0E-03	3.0E-02	7.8E-03	64	54	cytokinesis
GO:0006928	−0.49	1.0E-02	5.9E-03	7.8E-03	97	60	cellular component movement
GO:0001666	−0.31	1.4E-02	7.4E-03	1.0E-02	153	115	response to hypoxia
GO:0000922	−0.39	7.0E-03	1.6E-02	1.1E-02	86	77	spindle pole
GO:0031965	−0.33	1.7E-02	8.6E-03	1.2E-02	162	108	nuclear membrane
GO:0005813	−0.21	2.6E-02	7.4E-03	1.4E-02	353	165	centrosome
GO:0004674	−0.23	1.7E-02	1.3E-02	1.5E-02	374	217	protein serine/threonine kinase activity
GO:0070374	−0.36	3.1E-02	9.1E-03	1.7E-02	97	57	positive regulation of ERK1 and ERK2 cascade
GO:0044325	−0.37	9.1E-03	3.5E-02	1.8E-02	71	87	ion channel binding
GO:0055037	−0.63	1.5E-02	2.7E-02	2.0E-02	60	37	recycling endosome
GO:0004843	−0.46	1.8E-02	2.9E-02	2.3E-02	55	39	ubiquitin-specific protease activity
Negative predictors							
GO:0051436	0.55	2.5E-02	1.0E-03	5.1E-03	65	27	negative regulation of ubiquitin-protein ligase activity involved in mitotic cell cycle
GO:0045666	0.15	4.0E-01	3.6E-03	3.8E-02	68	50	positive regulation of neuron differentiation
GO:0042393	0.17	3.6E-01	7.1E-03	5.0E-02	62	47	histone binding

**β**: the regression coefficients estimated by the Cox-PH model. **CP**: the composite p-value, which is the square root of the product of the Log-rank test p-value and the corresponding Cox-PH p-value. **N1**: the number of member genes covered by the corresponding MMS or GO term. **N2**: the number of mutated member genes present in at least one training sample. Note that a single gene can be covered by more than one GO term.

We addressed the multiple-testing problem in the identification of predictive MMSs for patient survival by calculating false discovery rate (FDR) with a permutation-based algorithm (see Material and Methods section). In the implementation, we considered not only the skewness of the distribution of the effect parameters estimated from the original datasets (Figure 1C), but also the asymmetry of their null distribution established from the randomly permutated datasets (Figure S1). Because only one negative predictor (MMS) was rigorously selected, the analysis was focused on the 19 MMSs associated with good clinical outcomes. The result showed that, when those MMSs are stated to be significant, the FDR could be controlled at the level of 0.15.

Based on the definitions, we partition the cognate GO terms of the 19 positive predictors (MMSs) into six groups: (1) the gene products (proteins) locate in the cell organelles (membrane) responsible for waste disposal (lysosome) and material transport (recycling endosome); (2) the gene products locate in the sub-cellular structures playing roles in mitosis (nucleosome, spindle pole, centrosome); (3) the gene products perform function in cell division (mitosis and cytokinesis); (4) the gene products are involved in cellular responses to nutrient and hypoxia; (5) the gene products play roles in cancer pathways (integrin-mediated signaling pathway and positive regulation of ERK1 and ERK2 cascade); and (6) others. Numerous records regarding those GO terms' relevance to the formation, progression and therapy of tumors can be located in the literature (see Discussion section).

**Figure 2:**
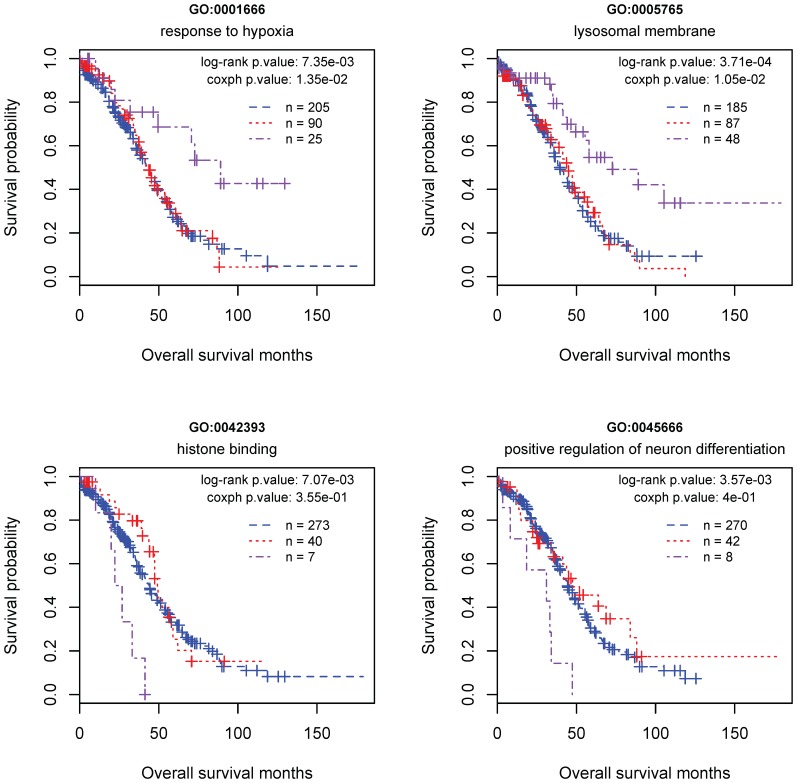
The illustration of the dose-dependent effect of somatic mutations on survival outcomes. Each plot demonstrates the relationship between the overall survival months and a specific macro mutation signature (MMS) that corresponds to a GO term. The purple curve represents the patients each of whom has at least two somatic mutations on the member genes of the indicated MMS (i.e., GO term). The red curve represents the patients each of whom has one somatic mutation on the member genes of the indicated MMS. The blue curve represents the patients without any somatic mutation on the member genes of the indicated MMS.

By looking into the Kaplan-Meier survival curves, we found a “dose-effect” relationship between the somatic mutations and survival outcomes. That is, for a specific GO term, a single mutation on the member genes usually does not make much difference to the patient survival time but two or multiple mutations do (Figure 2).

### Robustness analysis of the selected predictors

In order to test the robustness of our main result, we randomly split the 320 training samples into two equal-size subsets, and estimated the effects of the 22 predictive signatures on each subset separately. The result showed that the sign (positive or negative) of the estimated regression coefficients of hazard functions on the MMSs were consistent with those estimated using the entire training set (upper left plot of Figure S2).

We further tested if each of the 22 selected MMSs can individually predict the survival of cancer patients in the validation set. The results showed that, just the predictors ranked at the second, third and fifth places had a marginally significant p-value (upper right and bottom plots of Figure S2). While this analysis only provided a minor support to our findings in the last subsection, the result is aligned to our expectation. This is because the insufficiencies of the training set, i.e. the small sample size (N = 140) and un-validated mutations, could lead to a lower statistical power.

### A classification tree model for patient survival prediction

The findings presented above inspired us to build a classification tree to predict the patient survival using the 22 identified MMSs. More specifically, based on the measures of all three negative predictors, we can separate a poor-prognosis group from the entire set of training samples whose members meet 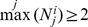, where 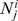 indicates the value of the *j^th^* negative MMS on the *i^th^* sample. Then, based on the values of the top *k* positive predictors and the same criterion, i.e. 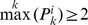 with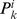 indicating the value of the *j^th^* positive MMS on the *i^th^* sample, a good-prognosis group can be split from the remaining samples that constitute an intermediate-prognosis group. See Figure S3 for an illustration. The threshold for the combined MMS values in the partition was heuristically chosen according to the pattern of dose-dependent effect of several MMSs of high interest to patient survival, as showed in Figure 2 and described in the ending paragraph of the first result subsection.

**Figure 3:**
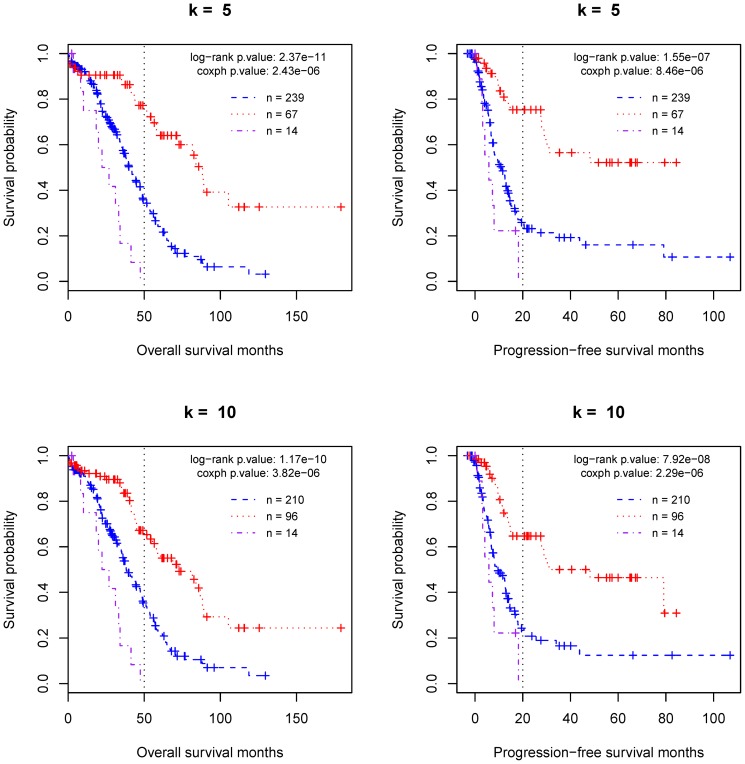
The classification of the training set of ovarian cancer patients by the proposed tree model. In each plot, the considered predictors include all three negative MMSs and the most significant (or top) *k* (5 or 10) positive MMSs as summarized in Table 1. The purple, red and blue curves represent the predicted poor, good, and intermediate-prognosis groups, respectively.

As shown in Figure 3, the patient groups generated by the tree model are significantly differentiated with respect to the times of overall survival (OS) and progression-free survival (PFS). Regardless of the *k* value (5 or 10), the Log-rank test p-value is less than 1.2×10^−10^ for OS and is less than 1.6×10^−7^ for PFS. From the Kaplan-Meier survival curves, we found that, for the poor-prognosis group, the upper limits of OS and PFS are 50 and 20 months, respectively. They are also the time points when the differences in the survival probabilities between the good-prognosis group and intermediate prognosis group become sharper. It is worth noting that, the choice of *k* value is somewhat arbitrary. The value determines the size of the predicted good-prognosis group that has a better survival curve compared to the intermediate-prognosis group. Therefore, a prior knowledge about the proportion of good-prognosis samples would help with the specification of *k* value.

### Model validation using independent datasets

**Figure 4:**
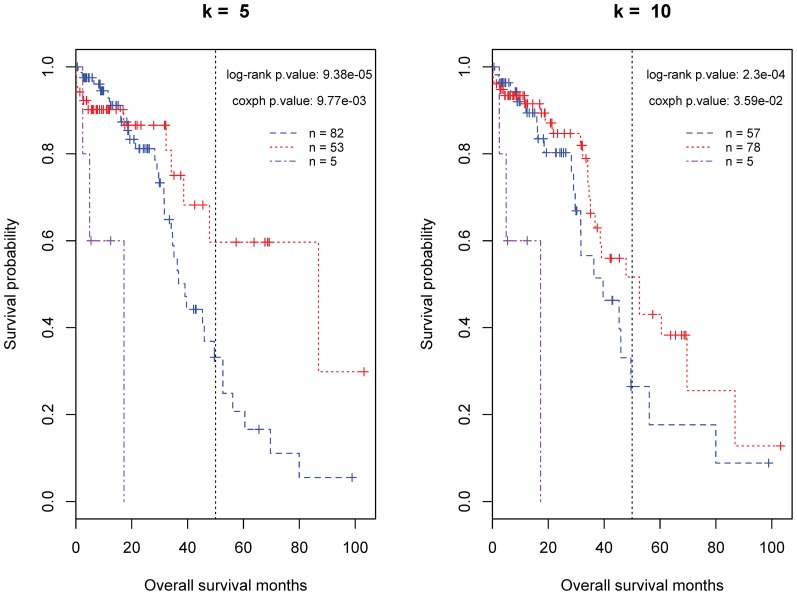
The classification of the validation set of ovarian cancer patients by the proposed tree model. In each plot, the considered predictors include all three negative MMSs and the most significant (or top) *k* (5 or 10) positive MMSs as summarized in Table 1. The purple, red and blue curves represent the predicted poor, good, and intermediate-prognosis groups, respectively.

We validated the tree model by applying it to an independent dataset of Ov-HGSCs. As shown in Figure 4, the survival curves of the patients in the three (good-, poor- and intermediate-prognosis) groups resemble those observed for the training set. The group effect on overall survival time is significant (Log-rank test p-value <0.001). When k is 5, the good prognosis group in this validation set has the same OS survival probability (∼30%) as that in the training samples. Moreover, interestingly, although the underlying negative predictors are not defined on a stringent statistical criterion, both the survival profile of the poor-prognosis group and the patient percentage (5/140 = 3.8%) of in this group are similar to those (14/320 = 4.5%) of the training set. It is worthy of note that, in the TCGA database, the observed somatic mutations of the samples in the validation set have not been confirmed by other methods yet. The average number of mutations in this set is approximately 80, much higher than those (∼50 observed and ∼46 validated) of the training set. Hereby, the classification results are more sensitive to the number of used predictors.

**Figure 5:**
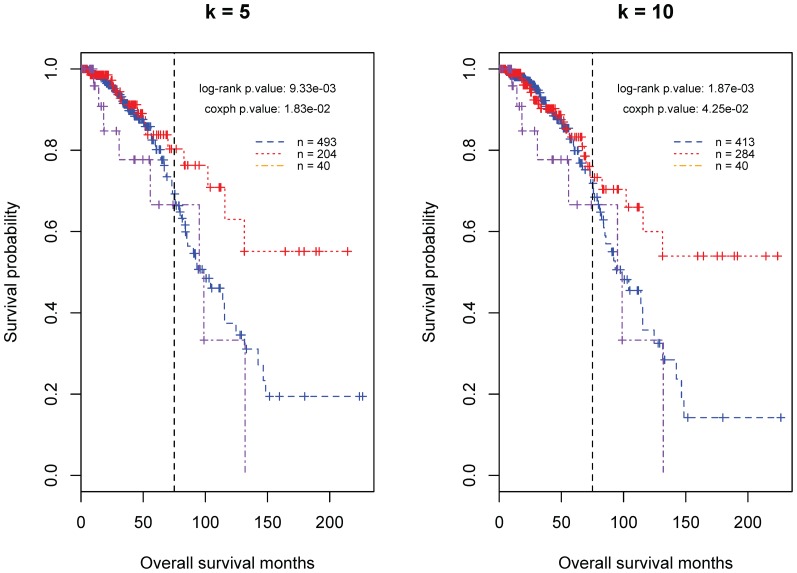
The classification of breast cancer patients by the proposed tree model. In each plot, the considered predictors include all three negative MMSs and the most significant (or top) *k* (5 or 10) positive MMSs as summarized in Table 1. The purple, red and blue curves represent the predicted poor, good, and intermediate-prognosis groups, respectively.

Recent studies showed that the formation of ovarian tumors shares common cancer drivers with breast tumors. We assume that these two diseases may be similar regarding the biological mechanisms underlying the variance in the patient survival time. We look into this issue by applying the identified predictors for Ov-HGSCs to the TCGA data of invasive breast carcinomas. As shown in Figure 5, we can identify a good-prognosis group using the top positive predictors but cannot separate a poor-prognosis group via the negative predictors. The difference in the survival probability between the good-prognosis patents and other patients becomes evident at the point of 75 months, 25 months more than the time for ovarian carcinomas.

### Comparison between mutation signatures and expression signatures

**Figure 6:**
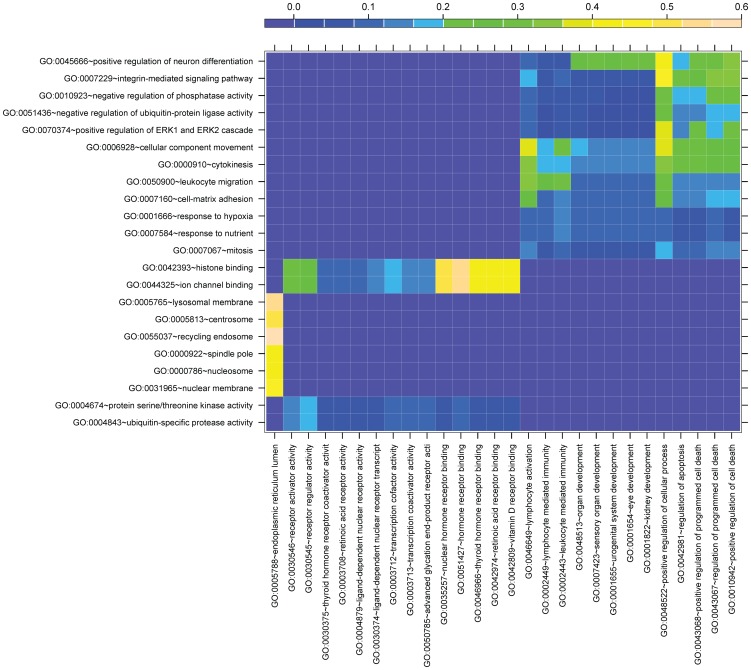
The visualization of the semantic similarity between the MESs and MMSs. The similarity is measured by a coefficient in the range of 0 to 1. 1 is the theoretical maximum of the similarity coefficient. For the GO term pairs considered here, the values are consistently less than 0.6.

By analyzing the TCGA clinical and mRNA expression data of Ov-HGSCs, we identified 333 expression predictors (genes) for the overall survival time of patients with the p-values less than 0.01. 28 functionally specific non-redundant GO terms, either at level-4 or level-5 as categorized by DAVID [19], were over-represented (FDR <0.1) by these genes. Hereafter, we named those 28 GO terms macro expression signatures (MESs). The matrix of the semantic similarity between the MESs and macro mutation signatures (MMSs), i.e. the GO terms corresponding to the 22 significant MMSs, was evaluated using the algorithm documented in [20]. As shown in Figure 6, the similarity coefficients are low in general. Four MES::MMS pairs have the coefficients over 0.5. They are: GO:0005788 (endoplasmic reticulum lumen) versus GO:0055037 (recycling endosome); GO:0005788 versus GO:0005665 (lysosomal membrane); GO:0051427 (human receptor binding) versus GO:00044325 (ion channel binding), GO:0051427 versus GO:0042393 (histone binding). Moreover, five MESs, relevant to the regulation of cellular process and cell death, show modest levels of similarity to seven MMSs, which correspond to some specific molecular functions and biological processes including integrin-mediated signaling pathway (GO:0007229) and positive regulation of ERK1 and ERK2 cascade (GO:0070374). These results suggest that: (1) only several survival-relevant somatic mutations impact the clinical outcomes via the modification of the expression level of the host genes; and (2) the proteins located on the cell organelles responsible for material transport and waste disposal may be crucial for the survival of cancer patients in that both the modification of properties (due to a non-synonymous mutation) and the change of expression level in cancer cells can significantly influence the clinical outcomes.

## Discussion

Over the last few decades, cancer researchers have pinpointed hundreds of cancer genes [21], [22], including oncogenes and cancer suppressor genes, and established a number of DNA-alteration based theories for carcinogenesis [5], [23]. Nevertheless, the genetic determination of survival outcomes for patients with malignant tumors has been less investigated yet. By analyzing 320 ovarian tumor samples, we found that somatic mutations favorable to the patient survival are predominant in ovarian carcinoma compared to those indicating poor clinical outcomes. This observation highlights the vulnerability of cancer cells to “extra” mutations. That is, while the cancer-driver mutations prompt cancer cells to divide in an uncontrolled way or offer them selection advantage over the adjacent normal cells, the extra mutations may restrict the continuous proliferation in certain microenvironments. When the restriction occurs in some important organs such as liver and spleen, where ovarian metastases usually lead to mortality, the extra mutations may benefit the patient survival. Such a mechanism can be elucidated by a further scrutiny of our results. For example, among the predictive MMSs identified for good prognosis, there is one that measures the mutation events occurring on the genes involved in the biological process of “response to hypoxia” [24]. It is well known that the activation of anaerobic glycolysis (the Warburg effect) provides most of the building blocks required to duplicate the cellular components of a dividing cell; therefore, it is also essential for carcinogenesis [25], [26]. If the properties of one or multiple protein(s) involved in anaerobic glycolysis are altered, the tumors may lose the ability to produce enough energy for maintaining their growth. As a result, the carcinogenesis can be retarded.

On the other hand, in many cases, cancer cells acquire mutations to constitutively activate their survival pathway and to develop chemo-resistance. This mechanism seems to cast a shadow on our explanation to the main conclusion of this study. However, the dilemma could be resolved to some extent if we assume that only a few new driver mutations occur as the responses to the treatments. This assumption is supported by our preliminary analysis which showed that the average numbers of somatic mutations in Ov-HGSCs don't substantially increase across the development stages (from II to IV).

Our analysis suggests that the proteins located on the cell organelles responsible for material transport and waste disposal bear a special importance for cancer mortality since both the modification of properties (due to a non-synonymous mutation) and the change of expression level in cancer cells can significantly impact the clinical outcomes. In particular, the identified predictors for good clinical outcomes include the MMS corresponding to the cell component GO term “lysosomal membrane”. This result provides the genetic insight into and clinical support for a promising cancer therapy strategy, in which the lysomoses of cancer cells can be treated as the drug targets. The strategy arose from the perception that the altered lysosomal trafficking and increased expression of the lysosomal proteases termed cathepsins may form an “Achilles heel” for cancer cells by sensitizing them to death pathways involving lysosomal membrane permeabilization and the release of cathepsins into the cytosol [27], [28], [29], [30]. A recent study on the screening of a small molecule drug library provided strong evidence for this mechanism. The authors found that over half of the 11 compounds that induced significant cell death in p53-null colon cancer cells triggered lysosomal membrane permeabilization and cathepsin-mediated killing of tumor cells [31]. We speculate that these compounds may functionally resemble the mutations present on the genes related to lysosomal membrane. We further surmise that, for an ovarian cancer patient with a single mutation on the lysosomal membrane related genes, an additional functional disruption of these genes caused by the anti-cancer compounds (or by other treatments) may offer the patient a better chance for survival, which is similar to those patients denoted by the purple curve in the upper right plot of Figure 2.

Resistance to apoptosis and chemotherapy is a critical factor in cancer recurrence and patient relapse. Several studies over the last decade have demonstrated that ECM/integrin signaling provides a survival advantage to various cancer cell types against numerous chemotherapeutic drugs and against antibody/radiotherapy therapy [32], [33], [34]. Our result implies that such an advantage for cancer cells can be interrupted by the mutations occurred on the cognate genes. As shown in Table 1, the MMS corresponding to the biological process of “integrin-mediated signaling pathway” is a positive predictor for the survival time of Ov-HGSC patients. Furthermore, we find that the mutations on the genes that positively regulate ERK1/2 cascade [35] can be deleterious to the continuous proliferation of cancer cells in the sense that the patients with such mutations had a longer survival time. These observations suggest that even the mutations whose host genes play a role in a cancer pathway may benefit the survival of cancer patients.

Another novel finding from this study is the dose-dependent effect of somatic mutations on survival outcomes. In light of this observation, we established a classification tree model to predict the survival profiles of the Ov-HGSC patients. The model is robust and performs comparably to the classifiers created using gene expression and other –omic data [10], [11], [12], [13], [36]. The phenomenon that a single mutation does not make much difference to a biological process but two (or multiple) mutations do may be explained by genome evolution. That is, evolution often created “backup” genes (or gene fragments) that perform the normal functions of a specific gene (or gene fragment) and a biological aberration occurs only when both the gene (or fragment) and its backups are altered [37], [38]. In fact, this mechanism may explain why the formation and malignancy of cancer require multiple mutations. To clarify this point, it is worth noting that a lethal biological aberration for tumor cells can imply a favorable change for the cancer patients and vice versa.

The proposed classification method can be implemented in a flexible way. For example, using the MMS corresponding to the GO term “histone binding” as the only predictor, a group of seven patients whose overall survival time is consistently less than 50 months can be identified from the Ov-HGSC training samples (Figure 2: bottom left plot). Each of these poor-survival patients has a short list of “lethal” mutations. Specifically, for the first sample of this group, there are three lethal mutations present on the genes NOC2L, CHD8 and CHAF1B. For the other six samples, the host genes of the lethal mutations are (L3MBTL2, L3MBTL2, L3MBTL2), (UIMC1, RNF20), (UIMC1, RNF20), (HJURP, NCAPD2), (NASP, PKN1), (MCM2, NCAPD2), respectively. Among the eleven member genes, three have been identified as prognostic indicators of breast or gastric cancers in previous studies. The evidences include: overexpression of MCM2 in gastric tumors predicted poor prognosis in the patients [39]; knock down of HJURP reduced the sensitivity of breast cancer patients to radiation treatment [40]; the loss of CHD8 may be an indicator for biological aggressiveness in gastric cancer [41]. Another two, i.e. UIMC1and CHAF1B, are cancer-relevant. The former codes BRCA1-A subunit RAP80 [42], a protein important for genomic stability [43]. The latter codes the chromatin assembly factor-1/p60, a proliferation marker in various malignant tumors with prognostic value in renal, endometrial and cervical carcinomas [44]. Therefore, further investigation on the functions and interaction of the proteins coded by these genes may facilitate the inference of the personalized mechanisms for the mortality of ovarian carcinomas.

Recent studies found cancer-driving changes shared across tumor types [45]. A well-known hallmark is the genetic similarity between breast cancer and ovarian cancer. For example, the major driver genes BRCA1/2 for breast cancer are frequently (10∼20%) mutated in the cancer cells of the patients with ovarian tumors [7]. Moreover, somatic mutations on TP53 (a major cancer driver gene in Ov-HGSCs) have been observed in the breast cancer samples of all subtypes, including luminal A, B, basal-like, and Her2-enriched [46]. Interestingly, we found that the top predictive MMSs identified using the clinical data and SMS of Ov-HGSC can predict the survival time of breast cancer patients. However, the three predictors for poor-prognostic outcomes of ovarian cancer are invalid when applied to breast cancer. Intuitively, more significant predictive macro signatures for breast cancer could be identified using the information of the patients of the same disease but this work is out of our scope.

To date, survival prediction using the gene expression signatures for breast or ovarian cancer patients has been the subject of much research [47], [48], [49], [50], [51], [52], [53]. However, most of the reported predictive expression signatures cannot be consistently validated by the analysis on the independent datasets (cohorts) [54]. Our comparative analysis suggests that only a few survival-related somatic mutations impact the clinical outcomes by modifying the expression level of the host genes. A potential reason for the robustness deficiency in the expression-based prognostic signatures is the temporal and/or spatial gap between the sampling of the disease tissue and the occurrence of the lethal metastasis of cancer cells. We speculate that mutation prognostic signatures, such as those we identified, have an advantage over an expression-based signature in the sense that they are less likely subject to progression history and location transition of cancer cells.

At last, we note that there are some uncertainties in our results. First, a few genes (N = 16), such as FN1, are involved in both positive and negative predictors for patient survival. Those genes account for 1.6% (16/1016) of all the genes which have at least one mutation in the training set and are covered by the 22 significant MMSs. Second, the false discovery rate of the predictive MMSs is slightly high (at the level of 0.15 for the 19 MMSs associated with good clinical incomes). In other words, a small portion of those MMSs might be falsely identified. Nevertheless, these issues are relatively minor to affect our conclusions regarding the predominance of somatic mutations favorable to patient survival and the prognostic usefulness of the identified predictive MMSs as a whole.

## Material and Methods

### Somatic mutation dataset for Ov-HGSC training samples (Data-1)

The dataset of 321 tumor samples was generated from three *mat*-format files (version 2.4)in the TCGA database [9]. The archives containing these files are “*broad.mit.edu_OV.IlluminaGA_DNASeq.Level_2.100.1.0*”, “*hgsc.bcm.edu_OV.SOLiD_DNASeq.Level_2.1.6.0*”, and “*genome.wustl.edu_OV.IlluminaGA_DNASeq.Level_2.1.3.0*”, respectively. Among the total 16306 mutations identified by exome-sequencing, 14960 have been validated using other methods and were used in our study. Most validated mutations belong to four single nucleotide mutation categories, namely missense_mutation (68.09%), silence (21.39%), nonsense_mutation (4.26%) and splice_site (2.20%). Among them, 257 validated mutations occurred on the gene TP53 of 225 samples. The cancer samples contained in this dataset were also used in [7]. There is a trivial difference between the SMS analyzed in our study and that used in [8].

### Somatic mutation dataset for Ov-HGSC validation samples (Data-2)

This mat-format dataset (version 2.4) was obtained from the archive “*genome.wustl.edu_OV.IlluminaGA_DNASeq.Level_2.2.1.0*” at TCGA [9]. In total, there are 142 tumor samples and 11342 mutations, of which 111 are present on the gene TP53. None of these mutations has been validated yet. The mutation distribution over variant types is similar to that of the training set (Data -1). The entire mutation profiling was used in the study.

### Somatic mutation dataset for breast invasive carcinoma samples (Data-3)

The dataset containing 776 tumor samples [55] was downloaded from TCGA [9]. The corresponding *mat*-format file is located in the archive “*genome.wustl.edu_BRCA.IlluminaGA_DNASeq.Level_2.5.3.0*”. In total, there are 47243 mutations. The mutation distribution over variant types is similar to that of *Data-1*. Among these somatic mutations identified by exome-sequencing, only 6397 have been validated using other methods. The entire mutation profiling was used in the study.

### Clinical dataset for Ov-HGSC training samples (Data-4)

This dataset is contained in the supplement, “*Copy of TCGA-OV-Clinical-Table_S1.2.xlsx*”, of the TCGA paper [7]. We downloaded it from the Nature website. The dataset consists of the clinical information of 488 Ov-HGSC patients (samples), of which 320 had the somatic mutations collected in *Data-1*. This dataset was used because it contains the progression-free survival time (PFS) which are not present in the matrix data archive of [9]. While the tumor-stage and tumor-grade attributes are also available in the dataset, neither [7] nor our preliminary analysis showed that their effects on the survival time were statistically significant. Hereby, these two attributes were not considered as predictive variables in the study.

### Clinical dataset for Ov-HGSC validation samples

This dataset was downloaded from [9]. Out of 573 patients in this set, 140 had the somatic mutations collected in *Data-2*.

### Clinical dataset for breast invasive carcinoma samples

The dataset was downloaded from TCGA database. Out of 971 patients in this set, 737 had the somatic mutations collected in *Data-3*.

### GO dataset

The gene function annotation data for human was downloaded (on Oct 8, 2013) from The Gene Ontology (GO) website [56]. In the dataset, 18920 genes (symbols) were annotated to 13863 GO terms. We used a heuristic method to select the GO terms considered in this study. That is, a GO term was selected if the number of genes annotated to this term was between 50 and 500. The reason for doing so is twofold. First, if a GO term has only a few genes, the values of its corresponding MMS may be too sparse to perform an efficient statistical inference. Second, if there are too many genes annotated to a GO term, the functional category can be rather broad to infer meaningful biological insight from the results. While this setting was somewhat arbitrary, it won't introduce the selection bias that might substantially impact the conclusion.

### Gene expression dataset for Ov-HGSC training samples

The mRNA expression levels of the tumor sample contained in *Data-1* were measured on three different platforms, i.e. Affymetrix Human Exon1.0 ST Array, Agilent 244K Whole Genome Expression Array and Affymetrix HT-HG-U133A Array. In the study, the combined gene expression dataset of 11684 genes present on all three platforms was used. The dataset is a supplement of [7] and was downloaded from the Nature website.

### Methods for survival analysis

Survival analysis was performed using the statistical functions included in R package “survival” [57], [58]. For univariate survival analysis with a factorized MMS as the predictor, the function “*survdiff*” was implemented to generate the Log-rank test p-value. It worth noting that, when “*survdiff*” was applied to the breast cancer dataset in which the cases of death at an early stage are rare due to right censoring, we let the *rho* parameter equal to negative 2, *i.e.* assigned each death a weight of S(t)^−2^, where S(t) is the Kaplan-Meier estimate of survival. The Kaplan-Meier survival curves (in Figures 2, 3, 4, 5 and S1), with the censored observations being marked by a vertical tick, were obtained via the function “*survfit*”. Multivariate survival analysis was conducted using the function “*coxph*” which implements Cox PH regression.

### Identification of MMSs for survival prediction

We identified the predictive MMSs for overall survival time using the procedure presented in the Result section, and ranked them according to the composite p-value *CP*. The *CP* value for a MMS was calculated as the square root of the product of the p-values obtained from the Log-rank test and the corresponding Cox-PH analysis.

### Identification of expression predictors for survival time

The association between the patient survival time and the gene expression levels was evaluated by the Cox PH regression. Similar to the analysis for the association between a MMS and the survival time, the patient age at the initial diagnosis was included in the model as a covariate.

### Comparison between macro mutation signatures and expression signatures

The similarity matrix for the macro mutation signatures (MMSs) and macro expression signatures (MESs) was calculated by the function “*goSim*” in the R package “GOSemSim” [59]. In the employed method [20], the semantics of GO terms are encoded into a numeric format and the different semantic contributions of the distinct relations are considered.

### Estimation of FDR

By adapting the methods used in [60], [61], we developed a permutation-based algorithm to estimate the false discovery rate (FDR) for the 19 predictive MMSs associated with good clinical outcomes. First, we generated 500 shuffled datasets via randomly permutating the clinical records of the 320 training samples while keeping their mutation profile untouched. Then, we repeated the survival analysis by the same method used in the identification of predictive MMSs, and recorded the Log-rank p-values (p_rank_), Cox-PH p-values (p_cox_), the complex p-values (p_cp_) as well as the regression coefficients (i.e. the beta values *c*) for all the 562 addressed MMSs. By doing so, we established the null distributions for p_rank_, p_cox_, p_cp_ and *c*, respectively. Finally, we compared the true distributions of p-values and regression coefficients to the corresponding null distributions to estimate false discovery rate by the following equation.
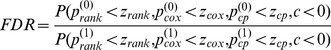
(1)


In (1), 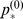 is a p-value from the null distribution and the subscript index * represents “rank”, “cox” or “cp”; 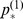 is a p-value from the true distribution; 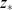 is the threshold specified for the identification of predictive MMSs, and it is set to be 0.05, 0.05 or 0.025 for p_rank_, p_cox_ or p_cp_, respectively. The numerator is the fraction of p-values from the null distributions that fall below the thresholds (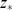) with the cognate regression coefficients less than 0. The denominator is the corresponding fraction for the estimates of p-values and regression coefficients based on the original dataset.

### Availability

R codes for the statistical analysis are available upon request.

## Supporting Information

Figure S1 **The asymmetry of the null distributions of the effect parameters**. The volcano plot of the Cox-PH p-values and regression coefficients for the 562 considered MMSs is based on the results of five randomly shuffled datasets.
(TIF)

Figure S2 **Robustness analysis of the predictive MMSs. Top-left**: The scatter plot shows the regression coefficients estimated from the two equal-size subsets of 320 training samples using the same Cox-PH model in the identification of the predictors. The solid squares (triangle) represent the 19 (1) MMSs which were rigorously selected and associated with good (poor) clinical outcomes. The solid circles represent the two MMSs which were selected in a less-rigorous way and were associated with poor clinical outcomes. The MMSs focused in the top right and bottom plots of this figure are marked with red. **Top-right (bottom-left, bottom-right)**: The results were obtained by analyzing 140 training samples. Each plot demonstrates the relationship between overall survival months and a specific macro mutation signature (MMS) that corresponds to a GO term. The purple curve represents the patients each of whom has at least two somatic mutations on the member genes of the indicated MMS (i.e., GO term). The red curve represents the patients each of whom has one somatic mutation on the member genes of the indicated MMS. The blue curve represents the patients without any somatic mutation on the member genes of the indicated MMS.
(TIF)

Figure S3
**An illustration of the proposed classification tree model for patient survival prediction.** This sample tree is generated using the three negative predictors (N_j,_ 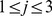) and five positive predictors (P_k,_ 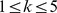) as the features. *S* represents the entire sample (or patient) set. *B* represents the predicted poor-prognosis set of patients. 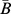
represents the remaining patient set after *B* is excluded. *G* represents the predicted patient set with good-prognosis. M represents the intermediate-prognosis set of patients, which is the remaining section of *S* after *B* and *G* are excluded. Note that in this sample tree, the feature tested at each internal node is a feature set instead of a single feature, which is different from the traditional classification/decision tree model.(TIF)

Table S1 **The genes involved in the GO terms corresponding to the predictive MMSs.**
(XLSX)
