# Supplementary figures and images for: Somatic Mutations Favorable to Patient Survival Are Predominant in Ovarian Carcinomas
Source: PLoS One. 2014 Nov 12;9(11):e112561. doi: 10.1371/journal.pone.0112561 (PMC4229214; doi:10.1371/journal.pone.0112561)

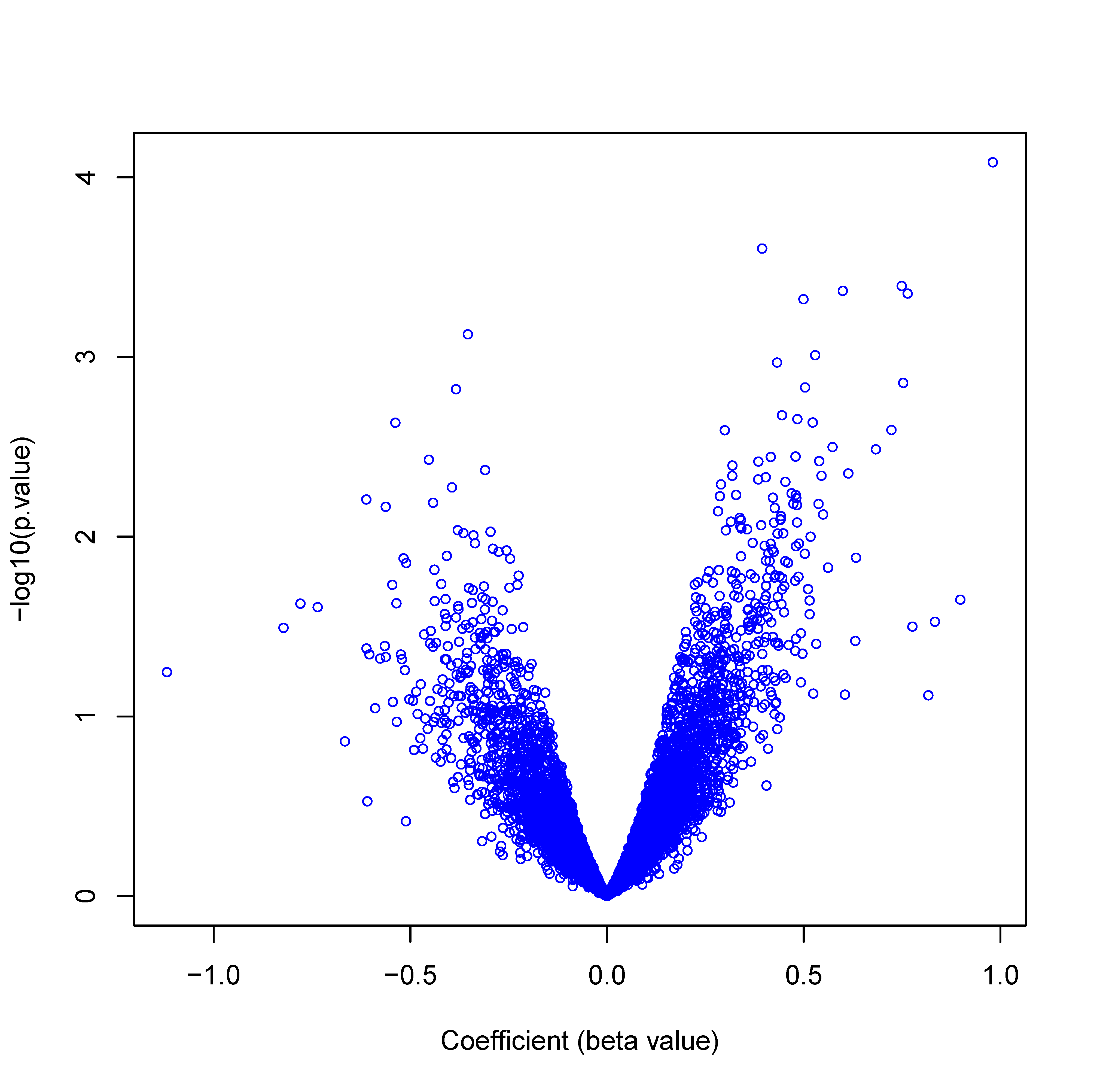

Supplement: Figure S1 — The asymmetry of the null distributions of the effect parameters. The volcano plot of the Cox-PH p-values and regression coefficients for the 562 considered MMSs is based on the results of five randomly shuffled datasets. (TIF) [file pone.0112561.s001.tif]

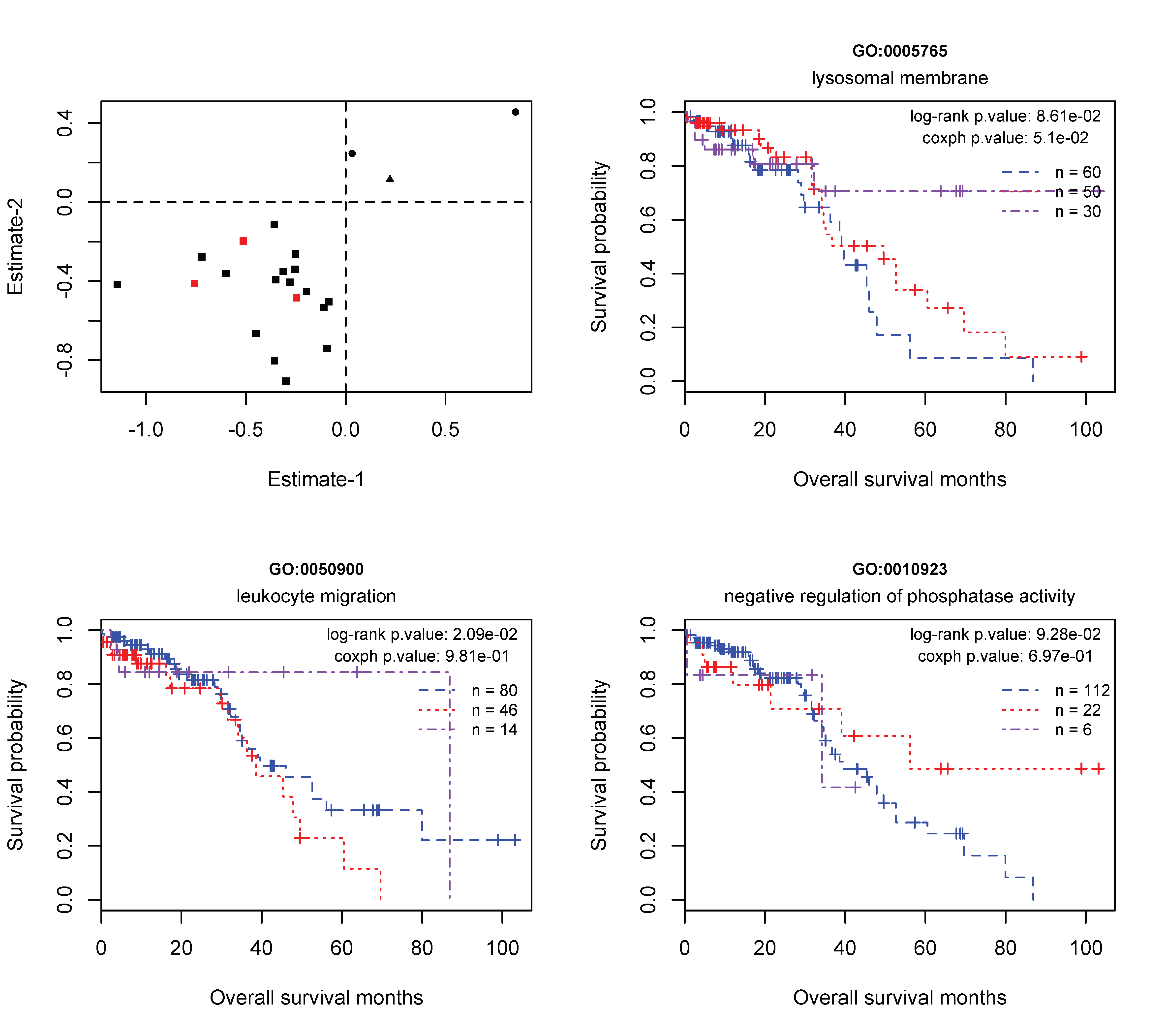

Supplement: Figure S2 — Robustness analysis of the predictive MMSs. Top-left: The scatter plot shows the regression coefficients estimated from the two equal-size subsets of 320 training samples using the same Cox-PH model in the identification of the predictors. The solid squares (triangle) represent the 19 (1) MMSs which were rigorously selected and associated with good (poor) clinical outcomes. The solid circles represent the two MMSs which were selected in a less-rigorous way and were associated with poor clinical outcomes. The MMSs focused in the top right and bottom plots of this figure are marked with red. Top-right (bottom-left, bottom-right): The results were obtained by analyzing 140 training samples. Each plot demonstrates the relationship between overall survival months and a specific macro mutation signature (MMS) that corresponds to a GO term. The purple curve represents the patients each of whom has at least two somatic mutations on the member genes of the indicated MMS (i.e., GO term). The red curve represents the patients each of whom has one somatic mutation on the member genes of the indicated MMS. The blue curve represents the patients without any somatic mutation on the member genes of the indicated MMS. (TIF) [file pone.0112561.s002.tif]

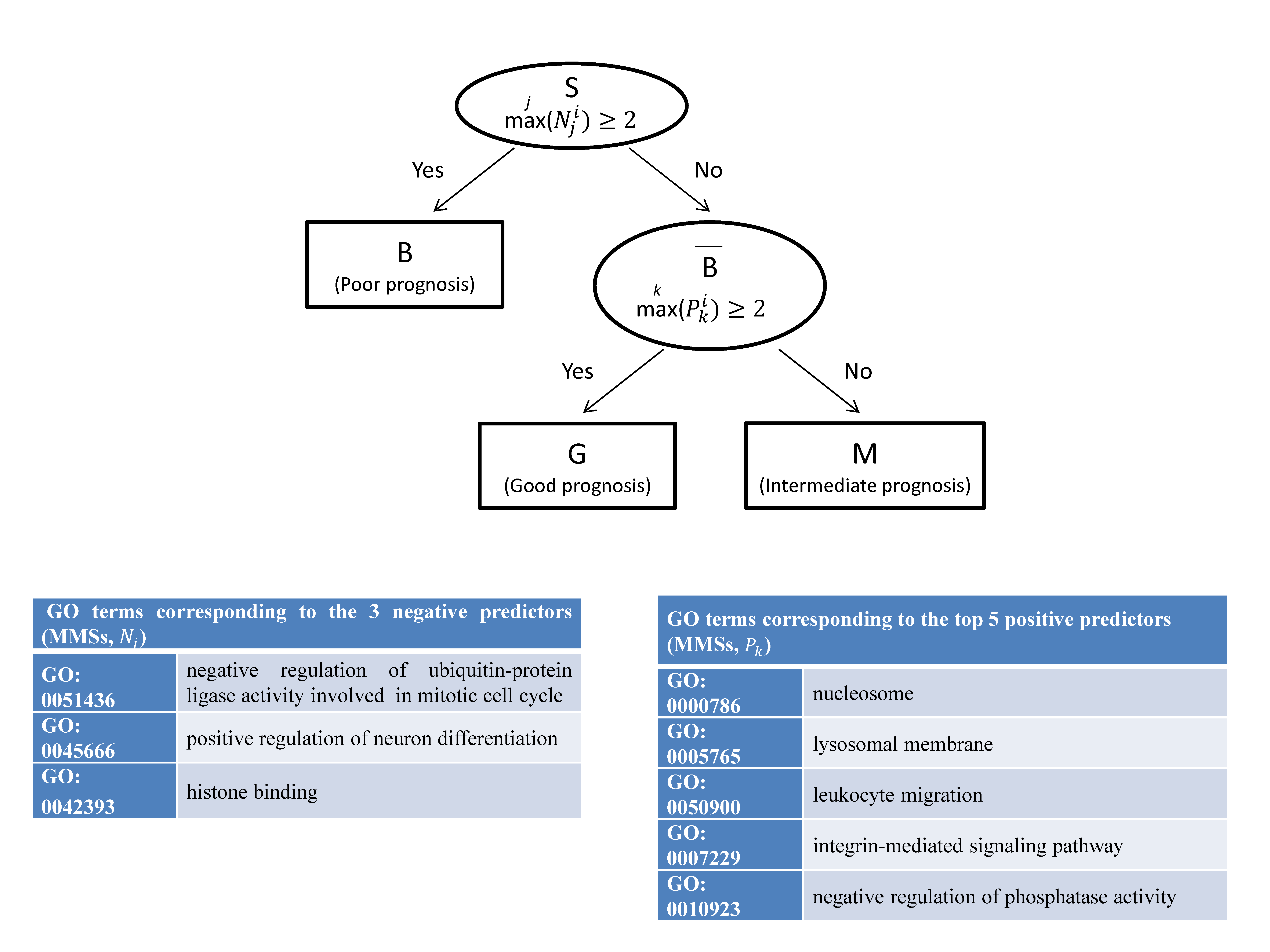

Supplement: Figure S3 — An illustration of the proposed classification tree model for patient survival prediction. This sample tree is generated using the three negative predictors (Nj, ) and five positive predictors (Pk, ) as the features. S represents the entire sample (or patient) set. B represents the predicted poor-prognosis set of patients. represents the remaining patient set after B is excluded. G represents the predicted patient set with good-prognosis. M represents the intermediate-prognosis set of patients, which is the remaining section of S after B and G are excluded. Note that in this sample tree, the feature tested at each internal node is a feature set instead of a single feature, which is different from the traditional classification/decision tree model. (TIF) [file pone.0112561.s003.tif]
